# Pembrolizumab Activity in Recurrent High-Grade Gliomas with Partial or Complete Loss of Mismatch Repair Protein Expression: A Monocentric, Observational and Prospective Pilot Study

**DOI:** 10.3390/cancers12082283

**Published:** 2020-08-14

**Authors:** Giuseppe Lombardi, Valeria Barresi, Stefano Indraccolo, Michele Simbolo, Matteo Fassan, Susanna Mandruzzato, Matteo Simonelli, Mario Caccese, Marco Pizzi, Arianna Fassina, Marta Padovan, Elena Masetto, Marina Paola Gardiman, Maria Giuseppina Bonavina, Maria Caffo, Pasquale Persico, Franco Chioffi, Luca Denaro, Angelo Paolo Dei Tos, Aldo Scarpa, Vittorina Zagonel

**Affiliations:** 1 Department of Oncology, Oncology 1, Veneto Institute of oncology-IRCCS, 35128 Padua, Italy; mario.caccese@iov.veneto.it (M.C.); marta.padovan@iov.veneto.it (M.P.); vittorina.zagonel@iov.veneto.it (V.Z.); 2 Department of Diagnostics and Public Health, Section of Pathology, University of Verona, 37134 Verona, Italy; valeria.barresi@univr.it (V.B.); michele.simbolo@univr.it (M.S.); aldo.scarpa@univr.it (A.S.); 3 Immunology and Molecular Oncology Unit, Veneto Institute of Oncology—IRCCS, 35128 Padua, Italy; stefano.indraccolo@iov.veneto.it (S.I.); ariannafassina@yahoo.it (A.F.); elena.masetto@iov.veneto.it (E.M.); 4 Department of Medicine (DIMED), Surgical Pathology and Cytopathology Unit, 35128 Padua, Italy; matteo.fassan@unipd.it (M.F.); marco.pizzi.1@unipd.it (M.P.); marinapaola.gardiman@aopd.veneto.it (M.P.G.); angelo.deitos@unipd.it (A.P.D.T.); 5 Department of Surgery, Oncology and Gastroenterology, University of Padova and IOV-IRCCS, 35128 Padova, Italy; susanna.mandruzzato@unipd.it; 6 Department of Biomedical Sciences, Humanitas University, 20089 Milan, Italy; matteo.simonelli@hunimed.eu (M.S.); pasquale.persico@hunimed.eu (P.P.); 7 Medical Direction Unit, Veneto Institute of Oncology, IOV-IRCCS, 35128 Padua, Italy; mariagiuseppina.bonavina@iov.veneto.it; 8 Department of Biomedical and Dental Sciences and Morphofunctional Imaging, Unit of Neurosurgery, University of Messina, 98122 Messina, Italy; mcaffo@unime.it; 9 Neurosurgery Unit, Azienda Ospedaliera di Padova, 35128 Padua, Italy; franco.chioffi@aopd.veneto.it; 10 Academic Neurosurgery, Department of Neurosciences, University of Padua, 35128 Padua, Italy; luca.denaro@unipd.it

**Keywords:** pembrolizumab, mismatch repair, high grade glioma, glioblastoma, TMB

## Abstract

Introduction: Pembrolizumab demonstrated promising results in hypermutated tumors of diverse origin. Immunohistochemical loss of mismatch repair (MMR) proteins has been suggested as a surrogate of hypermutation in high-grade gliomas (HGG). We evaluated the efficacy and safety of pembrolizumab in relapsing HGGs with immunohistochemical loss of at least 1 MMR protein. Molecular biomarkers of pembrolizumab activity were also analyzed. Methods: Consecutive patients with recurrent HGG and partial or complete loss of MMR protein expression were prospectively enrolled; they received pembrolizumab 200 mg once every 3 weeks until disease progression. The primary endpoint was disease control rate (DCR). Post hoc exploratory analyses included next-generation sequencing to assess tumor mutational burden (TMB), and immunostaining for CD8+ T-cells and CD68+ macrophages. Results: Among 310 HGG patients screened, 13 cases with MMR loss were enrolled: eight glioblastoma, four anaplastic astrocytoma, and one anaplastic oligodendroglioma. Median age was 43 years. DCR was 31%: four patients had stable disease and no patient had complete or partial response. TMB ranged between 6.8 and 23.4 mutations/megabase. Neither TMB nor gene mutations, nor CD8+ T-cell and CD68+ macrophage content, were associated with pembrolizumab activity. Conclusions: pembrolizumab showed no apparent benefit in these patients. No molecular biomarker was found to be associated with pembrolizumab activity.

## 1. Introduction

Anti-programmed death 1 (PD-1) immune checkpoint inhibitors have shown efficacy in patients with several different cancer types. These new drugs can overcome T cell inhibition and promote an immune response against the tumor [1]. Several markers including PD-L1 expression, lymphocytic infiltrates, tumor mutational burden (TMB), and mismatch repair deficiency (MMRd) appear to be helpful in predicting response to immune checkpoint inhibitors in specific tumor types [2]. In particular, recent studies showed that MMRd tumors have 10 to 100 times more somatic mutations than MMR-proficient cancers and that this hypermutation state could lead to cumulative neoantigen generation, which can activate the immune system and promote tumor destruction [3]. Immunohistochemistry for the DNA MMR proteins MSH2, MSH6, MLH1 and PMS2 is already used to screen for MMRd in the spectrum of cancers belonging to Lynch syndrome [4]. In early 2017, Hodges et al. [5] reported that the immunohistochemical loss of at least one MMR protein was associated with hypermutation in gliomas, and this was confirmed in further recent studies [6,7,8].

Nivolumab and Pembrolizumab, two anti PD1 immune checkpoint inhibitors, demonstrated therapeutic efficacy in solid tumors with MMRd [2,9,10,11,12]. Based on these results, the US Food and Drug Administration (FDA) approved pembrolizumab for the treatment of MMR-deficient solid tumors. A few prospective clinical trials addressed the activity of immune checkpoint inhibitors in HGG. In prior randomized phase III studies (Checkmate 498 and Checkmate 143), Nivolumab was evaluated in newly diagnosed MGMT-unmethylated and recurrent glioblastoma patients and failed to extend overall survival compared to standard treatment [13,14]. Likewise, in a phase 1b trial (KEYNOTE-028), pembrolizumab showed poor results in patients with recurrent PD-L1 positive glioblastoma [15]. Unfortunately, in these studies, neither MMR status nor TMB were analyzed.

To better clarify the role of MMR status as a potential biomarker of pembrolizumab activity, we performed an observational pilot study, in which pembrolizumab was administered in recurrent HGG patients with loss of expression of MMR proteins, used as a surrogate marker for hypermutation.

## 2. Results

### 2.1. Patients and Treatment

At the Veneto Institute of Oncology-IRCCS, between May 2017 and May 2019, 310 patients with HGG were screened by IHC for MMR protein expression. In 260 (84%) cases, the analysis was done on the primary tumor, and in 50 (16%) on the recurrent tumor.

Thirty-seven HGG (12%) had the immunohistochemical loss (partial or complete) of at least 1 MMR protein; among these, 17 had an Eastern Cooperative Oncology Group (ECOG) Performance Status (PS) >2 and 7 were taking dexamethasone >4 mg/day. Therefore, 13 patients were finally enrolled and treated with pembrolizumab. Eight patients had a glioblastoma, four had an anaplastic astrocytoma and one had an anaplastic oligodendroglioma. Nine tumors (69%) were *MGMT* methylated and four (31%) *IDH* mutated.

**Figure 1:**
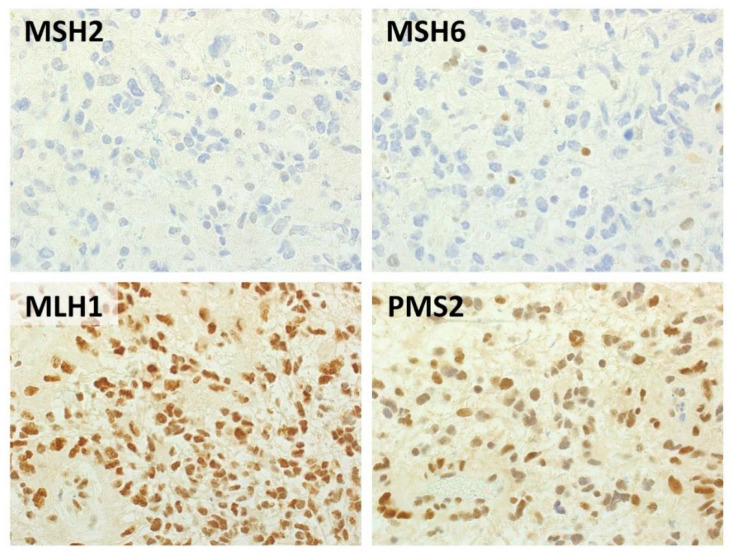
Representative histological features of a MSH2/MSH6-negative glioblastoma. Immunohistochemical analysis highlighted negativity for MSH2 and MSH6 in neoplastic cells, with preserved expression of MLH1 and PMS2. Background inflammatory cells were used as positive internal controls. (Immunoperoxidase stain, original magnification 40×).

Six HGGs had concurrent partial loss of MSH2 and MSH6, one had complete loss of both MSH2 and MSH6 (Figure 1); one had complete loss of MSH6 alone, two had partial loss of both MLH1 and PMS2, two partial loss of MSH2 alone and one partial loss of MSH6 alone. (Table 1; Table S1 and Table S2 in Supplementary Materials). In four cases (three glioblastomas, one anaplastic astrocytoma), IHC and molecular analyses were carried out on the primary tumor, and in nine cases on the recurrent tumor.

Median prior chemotherapy lines were two (range 1–5; 7/13 [54%] patients with two prior lines of chemotherapy) and all patients had received temozolomide and radiation therapy as first line treatment.

### 2.2. Clinical Activity and Tolerability

At the time of analysis, median follow up was 20.6 months. Patients were treated for 1 to 23 cycles of pembrolizumab (median, 3 cycles). All patients discontinued pembrolizumab due to disease progression; two patients were still alive.

The DCR was 31%: four patients had stable disease and no patient had complete or partial response. Nine patients (69%) showed progressive disease (Table 2).

Median duration of stable disease was 7.7 months (range 5.2–16.7). The four patients with stable disease were two with anaplastic astrocytoma, one glioblastoma and one anaplastic oligodendroglioma. Two cases had partial loss of both MSH2 and MSH6 protein expression, one had partial loss of MSH2 alone and one had partial loss of MSH6 alone. *IDH* was mutated in only one of these four tumors (Table S2 in Supplementary Materials).

**Figure 2:**
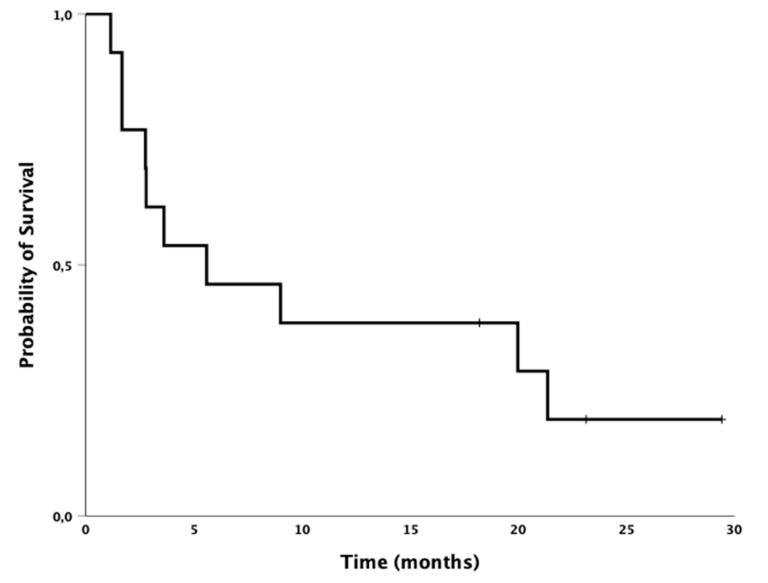
Kaplan–Meier survival analysis. Median overall survival for all patients (*n* = 13) was 5.6 months (95% CI 0.1–11.9) from start of pembrolizumab treatment.

Median PFS was 2.2 months (95% CI 1.6–2.8); the 6-month PFS was 23%. Median OS for all patients was 5.6 months (95% CI 0.1–11.9); the 12-month OS was 38% (Figure 2 and Table 3).

Therapy was well tolerated and only one patient (8%) reported a grade 3 adverse event (rash maculo-papular).

### 2.3. Molecular Characteristics

A series of post hoc exploratory analyses were performed on gliomas of 12 patients with available tumor tissue. For one patient, tissue was not available for molecular analyses.

#### 2.3.1. Multigene Mutational Status

**Figure 3:**
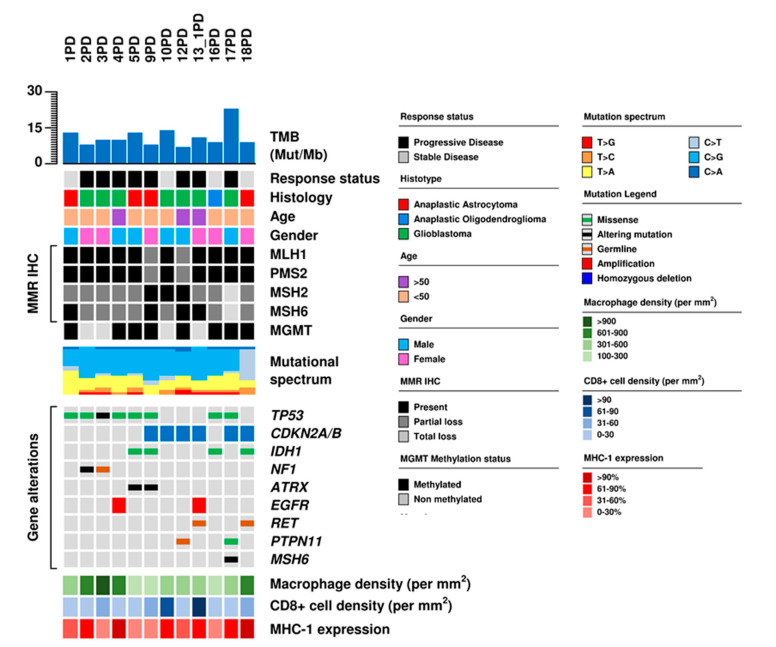
Genomic and immunological characteristics of 12 high-grade gliomas. The matrix shows the characteristics of each patient and matched tumor sample. Gene mutations, immunohistochemical analysis of genes involved in mismatch repair (MMR IHC), are correlated to histology, treatment response, tumor mutational burden (TMB) and immunological characteristics.

Average sequencing coverage obtained with tumor mutational load (TML) next generation sequencing (NGS) panel was 277× (120–556×) in tumor and 274× (125–651×) in normal samples. A total of 29 mutations in 14 genes were identified (Figure S1 in Supplementary Materials). Mutations were found in at least one gene in all 12 cases. The most frequent somatic mutations were in *TP53* (8/12; 67%) and *IDH1* (4/12; 33%) (Figure 3 and Table S3 in Supplementary Materials). *ATRX*, *NF1*, *PTPN11* and *RET* mutations were found in two cases (17%).

Mutations in *TP53*, *IDH1*, *ATRX* and *NF1* were confirmed by using the ACC GBM (Alleanza Contro il Cancro-Glioblastoma) capture-based custom panel.

A somatic truncating mutation was found in the *MSH6* MMR gene in GBM #17PD and it was confirmed by using the ACC GBM capture-based custom panel. This patient had progressive disease during pembrolizumab treatment.

Five patients had germline mutations: one had *PTPN11* Asp61Tyr (rs397507510) mutation, reported as pathogenic in the ClinVar database; one had *RET* Arg982Cys (rs17158558) mutation; three had a mutation in *NF1* (rs769087878), *PMS1* (rs2066459) and *RET* (rs149238501), respectively (these mutations are classified as of uncertain clinical significance in the ClinVar database).

Evaluation of tumor mutational burden, microsatellite instability and immunological characteristics of the tumor microenvironment

TMB in the 12 GBMs ranged between 6.7 and 26.9 (median: 10.02) (Figure 3, Table 2). A total of 8/12 cases (67%) had >9 muts/Mb and were considered hypermutated [7,16]. All tumors were microsatellite stable, and only case, #17PD, had a pathogenic *MSH6* somatic mutation. This latter tumor had the highest TMB (26.9 mutations/megabase), a CD8+ density of 30.6/mm^2^ and a macrophage density of 483.1/mm^2^.

**Figure 4:**
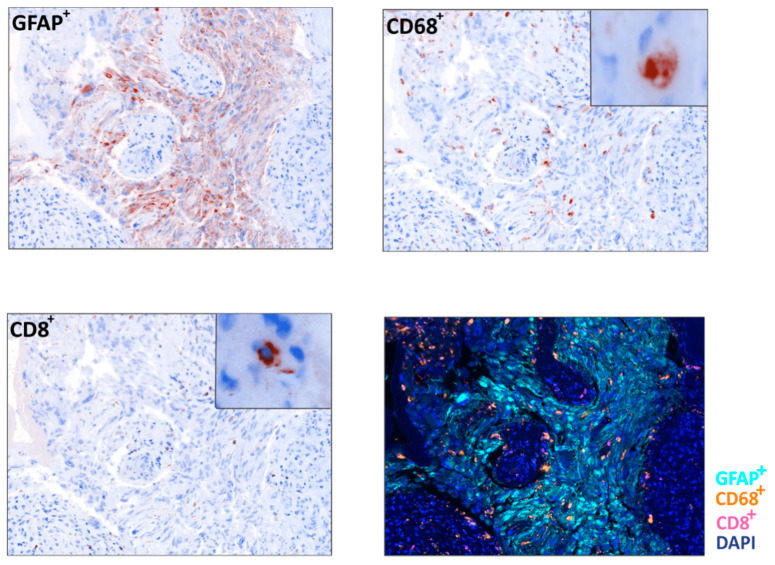
Immune infiltrate analysis in a glioblastoma sample. Representative example of a *Multispectral Imaging* field obtained from a glioblastoma sample in which the markers GFAP (light blue), CD68 (orange) and CD8 (pink) are reported (20× zoom, lower right panel) and relative pseudo-immunohistochemistry representations of the single cell populations (40× zoom). DAPI (blue) was used as nuclear counterstain.

Overall, the median value of TMB was 10.02 and 11.36 mutations/megabase, the median value of MHC-1 expression was 60% and 55%, the median value of macrophage density was 438.05/mm^2^ and 407.45/mm^2^, and CD8+ density was 25.9/mm^2^ and 30.05/mm^2^ for the PD and SD groups (Figure 4). All of these values as well as the complete or partial loss of MMR protein expression were not significantly different between patients with PD and SD (Table S4 in Supplementary Materials); thus, no molecular or microenvironmental factor was predictive of pembrolizumab efficacy.

#### 2.3.2. Gene and Chromosomal Copy Number Alterations

The CNV status was estimated for all 409 genes using sequencing data and, based on the chromosomal position of each gene, the status of chromosome arms was inferred. Seven genes had focal gene amplification: *EGFR* in two cases; *PDGFRA*, *CDK4* in four cases (10%); *KIT*, *KDR* and *MDM4* and *PIK3C2B* in one case. Two genes showed homozygous deletion: *CDKN2A* and *CDKN2B* in 6/12 cases (50%). The most frequent whole chromosome alterations were gains of chromosomes 7 and 8 (2/12; 17%) and loss of chromosome 9 (6/12; 50%).

## 3. Discussion

In this observational and prospective study, the administration of pembrolizumab in recurrent HGG showing MMR protein loss did not result in an apparent clinical benefit.

In previous prospective studies testing pembrolizumab or nivolumab in treatment-refractory HGG patients, MMR status had not been assessed. The phase 3, Checkmate 143 trial [14] showed a disease control rate of 29.4% (CR in 1.3% patients, PR in 6.5%, SD in 21.6%), 6-month PFS of 15.7% and 12-months OS of 41.8%, in patients with recurrent glioblastoma treated with nivolumab. The phase 1b, KEYNOTE-028 [15] trial evaluated safety and efficacy of pembrolizumab in 25 patients with PDL-1-positive recurrent glioblastoma and 31% of patients had already received two or more lines of chemotherapy. The DCR was 52% (PR 4%, SD 48%), 6-month PFS was 44% and 12-month OS was 74%. Finally, a very recent phase 2 study (KEYNOTE-158) [11] analyzed pembrolizumab efficacy in patients with various types of noncolorectal cancers, which were classified MSI-H/dMMR on the immunohistochemical loss of at least one MMR protein, or on the presence of MSI at PCR. Among all patients, only those with brain tumors, the histotype of which was not mentioned, did not show radiological response. The median PFS and OS of 1.1 (95% CI 0.7–2.1) and 5.6 (95% CI 1.5–16.2) months, respectively, were very similar to those observed in our patients. In patients with other tumors, the ORR (CR + PR) ranged between 18% and 57%. All these data sustain our conclusion that MMR immunohistochemical loss can not be used as a predictive biomarker of anti-PD1 efficacy in patients with HGG.

Although all our cases had partial or complete loss of MMR proteins at immunohistochemistry, they were microsatellite stable. Touat et al. [17] recently demonstrated that, differently from colorectal carcinomas, gliomas with either complete or partial MMR loss may lack MSI. Indeed, by single cell whole genome sequencing, they demonstrated that MSI may be present in subclones, and therefore it may not be detectable by MSI assays. This might explain why tumors in this cohort had MMR loss but not MSI.

In our series, eight cases (67%) (three treatment naïve and five temozolomide-treated) were hypermutated (>9 muts/Mb) and none were ultra-mutated (>100 muts/Mb) [7]. Hypermutation may be related to temozolomide treatment in 5/8 hypermutated cases, in which TMB was assessed on recurrent tumor.

The association between MMR status and hypermutation is still unclear in gliomas [6,8,18]. The high frequency of hypermutated gliomas in our cohort, which is much higher than the 3.5% previously reported in glioblastoma [5], may suggest that the immunohistochemical loss of MMR proteins is useful to detect hypermutated tumors, as recently proposed by McCord et al. [8]. Although 8/9 hypermutated gliomas in McCord et al. [8] had MMR immunohistochemical loss and *MMR* mutations, MSI status was not explored and whether hypermutated gliomas identified by means of MMR immunohistochemical loss are responsive to immune check point inhibitors remained unsolved. Our study showed that patients with hypermutated HGGs and immunohistochemical loss of MMR proteins had at best SD when treated with pembrolizumab, and we observed similar TMB median values in patients with progressive and stable disease. Since none of the tumors in our cohort had MSI and only one had an *MMR* mutation, our findings indicate that MMR immunohistochemical loss may be uncorrelated with MMR deficiency in gliomas and therefore it does not represent an optimal marker for selection of cases to be candidate to immune check point inhibitors.

Although the only case with concurrent complete loss of MSH6 and MSH2, associated with *MSH6* mutation, had the highest TMB (29.86 mut/Mb), the absence of MSI may account for the lack of response to pembrolizumab. Similarly, Touat et al. [17], in a recent retrospective analysis, showed no efficacy of anti-PD1 blockade in 11 patients with hypermutation and loss of MMR immunohistochemical expression. Therefore, although MMR immunohistochemical loss may be a surrogate of hypermutation, it is not predictive of response to anti-PD1 blockade in glioma patients.

An interesting study demonstrated that higher TMB was associated with improved survival in patients receiving immune checkpoint inhibitors across a wide variety of cancer types, with the exception of high-grade gliomas [19]. Noteworthy, the authors noted that the cut-off points to define “high TMB” markedly varied between tumor types and that for glioma was very low compared to those of other tumors (5.9/Mb versus 52.2/Mb in colorectal cancer). Therefore, we may hypothesize that HGGs may take benefit from ICI only if ultramutated, as reported in patients with MMR germline mutations [20,21].

Microenvironment characteristics may also be relevant in the response of HGGs to ICI. Similar to our study, a recent trial showed that, among 66 adult patients with recurrent GBM and treated with pembrolizumab or nivolumab, TMB was not different between responders and non-responders [22]. Interestingly, non-responder gliomas had an immunosuppressive expression signature and higher levels of CD68+ macrophage infiltration. In line with our previous study showing an enhanced immunosuppressive microenvironment in higher grade gliomas [23], patients with SD were those with lower grade gliomas and a GBM case with high mutational load, low presence of immune suppressive macrophages, high number of CD8^+^ T cells and high expression of MHC class I molecules. This observation suggests that a combination of high mutational load and low immune suppressive tumor microenvironment can underlie the response to immune checkpoint therapy in GBM.

Our finding that nonresponder HGG were poorly infiltrated with T cells but enriched in CD68+ macrophages further supports the suggestion that a combination of anti-PD1 and drugs targeting macrophages should be tested in HGG. Regorafenib, an oral multi-kinase inhibitor, could represent a valuable candidate drug to combine with anti-PD1, as it demonstrated the ability to inhibit tumoral macrophages [24] and improve OS in recurrent glioblastoma [25].

Our study has some limitations. First, we analysed a small cohort of patients; however, this is a monocentric, observational pilot study and MMR protein loss is rare in gliomas. Therefore, considerable effort was made to enrol this number of cases and perform molecular analyses. Second, our cohort was heterogeneous as it included cases with partial and complete loss of MMR proteins; However, based on prior observation [5], we had hypothesized that also cases with partial MMR loss could be hypermutated. Third, although we used an anti-PD1 drug, all our cases had either low or no PD-L1 expression; however, PD-L1 expression was not predictor of anti-PD1 efficacy in many trials analysing these agents in various types of tumors [26,27]; in particular, pembrolizumab showed limited benefit in selected patients with PD-L1 positive recurrent glioblastoma [15]. Fourth, the inclusion of both WHO grade III and IV gliomas made difficult the comparison with published data of phase 2 and 3 trials including only GBM patients. However, our results are similar to those of a prior retrospective study showing 1 PR, 2 SD, 7 PD and DCR of 28% in 25 recurrent, non-hypermutated HGGs including 10 grade III tumors, treated with pembrolizumab [28].

## 4. Materials and Methods

### 4.1. Patients

Thirteen patients were prospectively enrolled in this monocentric observational pilot study, which was approved by local institutional review board (IOV EC n. 6.18) and complied with International Ethical Guidelines for Biomedical Research Involving Human Subjects, good clinical practice guidelines, and the Declaration of Helsinki. Written informed consent was obtained for each patient. Inclusion criteria were: age ≥18 years, histologically confirmed diagnosis of HGG (anaplastic astrocytoma; glioblastoma; oligodendroglioma and anaplastic oligodendroglioma), relapse according to RANO (Response Assessment in Neuro-Oncology) criteria [29], evidence of complete or partial loss of at least 1 MMR protein assessed by immunohistochemistry at diagnosis or at recurrence, failure of both radiotherapy and temozomolide chemotherapy, no prior immunotherapy, ECOG Performance Status 0–2, and dexamethasone dosage ≤4 mg/day for 7 days prior to start of pembrolizumab. There was no limit on the number of prior treatment regimens, but chemotherapy had to be discontinued at least 4 weeks prior to start pembrolizumab. A further requirement was the presence of a bidimensionally measurable enhancing lesion with minimal diameters of 10 mm on magnetic resonance imaging (MRI). Eligibility also required adequate hematological, renal, hepatic function and absence of autoimmune diseases.

### 4.2. Procedures

Tumor MMR status was determined by immunohistochemistry to assess the expression of the four main components of the MMR complex (i.e., MLH1, PMS2, MSH2, MSH6; Dako, Glostrup, Denmark). Stained slides were jointly evaluated by two pathologists. For each MMR protein, the immunostaining was classified as: retained, complete loss, partial loss (defined as a heterogeneous pattern of staining with coexistence of positive and of at least 30% negative tumor cells).

Eligible patients received standard dosages of intravenous pembrolizumab 200 mg once every week until progression according to immunotherapy response assessment in neuro-oncology (iRANO) criteria [30] unacceptable toxicity, or patient decision. Baseline MRI was performed within 2 weeks of starting pembrolizumab. MRI for disease assessment was performed every 8 weeks or when clinically indicated. Adverse events were graded using National Cancer Institute Common Terminology Criteria for Adverse Events version 4.

### 4.3. Outcome

The primary endpoint was Disease Control Rate (DCR), defined as the proportion of patients with confirmed complete response (CR), partial response (PR) or stable disease (SD) per iRANO criteria in response to the treatment. Secondary endpoints included progression-free survival, defined as the time from the first dose of pembrolizumab to disease progression or death from any cause; overall survival, defined as the time from the first dose of pembrolizumab to the date of death from any cause; and duration of response, defined as the time from first documented evidence of complete/partial response or stable disease until the first documented sign of disease progression or death from any cause; and safety.

### 4.4. Post Hoc Exploratory Analyses

Methods for analyzing mutational and copy number variation status, tumor mutational burden and mutational signatures, target NGS sequencing, MGMT promoter methylation status, PD-L1 and MHC-1 expression, macrophage and CD8+ cell density and microsatellite instability have been reported in the Supplementary Materials (Methods S1).

### 4.5. Statistical Analysis

Progression-free survival (PFS), overall survival (OS) and duration of response/stability were evaluated using the Kaplan–Meier method. Patients who did not achieve a response or stability were excluded from the duration of response/stability analysis. Patients without a progression-free survival or overall survival event were censored at last assessment. Continuous variables were compared using the Mann–Whitney test; categorical variables were compared using Fisher’s exact test. The significance level was defined as *p* < 0.05 (SPSS 26).

## 5. Conclusions

In this observational, pilot and prospective study, we report a low activity of pembrolizumab in recurrent HGGs with partial or complete loss of MMR protein expression. Response to pembrolizumab did not correlate with any molecular and immunological characteristic of the tumor and its microenvironment. It is likely that the combination of anti-PD1 and drugs targeting CD68+ macrophages, such as regorafenib, could represent a valuable therapy to be tested.

## Figures and Tables

**Table 1:** Clinicopathological data of the 13 patients enrolled in the trial.

Patient	Age	Gender	ECOG PS	Histology	IDH Status	MGMT Status	Lines of Prior CT	Steroids at Baseline	MMR Protein Expression			
									MLH1	PMS2	MSH2	MSH6
1PD *	43	M	1	AA	wt	met	2	not	++	++	+-	++
2PD	47	F	1	GBM	wt	unmet	1	yes	++	++	+-	+-
3PD *	21	F	1	GBM	wt	unmet	2	yes	++	++	+-	+-
4PD *	55	M	1	GBM	wt	met	1	yes	++	++	+-	+-
5PD	34	M	1	AA	mut	met	4	not	++	++	+-	+-
9PD	30	F	1	AA	mut	met	3	yes	+-	+-	++	++
10PD	30	M	0	GBM	wt	unmet	1	not	++	++	++	+-
12PD *	65	M	2	GBM	wt	met	1	not	+-	+-	++	++
13_1PD	64	F	0	GBM	wt	unmet	1	not	++	++	+-	++
16PD	42	F	1	AODG	mut	met	5	not	++	++	+-	+-
17PD	37	M	1	GBM	wt	met	3	not	++	++	--	--
18PD	48	F	1	AA	mut	met	2	not	++	++	+-	+-
21PD	53	M	0	GBM	wt	met	1	yes	++	++	++	--

* In these cases, histological and molecular analyses were performed on primary tumors, in all other cases on recurrent tumors (all patients received temozolomide and radiation therapy as first treatment); ECOG PS: Eastern Cooperative Oncology Group Performance Status; M: male, F: female; GBM: glioblastoma; AA: anaplastic astrocytoma; AODG: anaplastic oligodendroglioma; wt: wild-type; mut: mutated; met: methylated; unmet: unmethylated; ++: marker expressed in the totality of tumor cells; +-: marker partially expressed in tumor cells; --: marker not expressed in tumor cells.

**Table 2:** Molecular data and outcome of the 13 patients enrolled in the trial.

Patient	Gene MMR Mutations	MSI	TMB (muts/Mb)	PD-L1 (% Tumor Cells)	MHC-1 (% Tumor Cells)	Macrophage Density (n/mm^2^)	CD8+ Density (n/mm^2^)	Response	PFS (Months)	OS (Months)
1PD *	none	absent	13.4	0	40	360.20	8.10	SD	5.29	9.00
2PD	none	absent	7.7	0	75	605.20	20.00	PD	1.12	1.68
3PD *	none	absent	9.78	0	30	971.20	45.40	PD	0.92	1.68
4PD *	none	absent	10.26	0	95	680.60	11.80	PD	2.37	3.61
5PD	none	absent	13.28	0	0	108.90	21.20	PD	1.54	29.40 °
9PD	none	absent	8.45	0	0	140.10	38.80	PD	0.69	1.15
10PD	none	absent	13.93	3	70	454.70	84.90	SD	7.36	19.98
12PD *	none	absent	6.76	0	50	328.00	0.00	PD	2.23	2.76
13_1PD	none	absent	10.84	5	70	393.00	90.70	PD	1.84	18.20 °
16PD	none	absent	8.97	0	0	256.79	3.75	SD	16.76	21.36
17PD	MSH6	absent	26.89	0	90	483.10	30.60	PD	2.14	2.79
18PD	none	absent	9.33	0	95	665.30	52.00	SD	8.11	23.13 °
21PD	n.d.	n.d.	n.d.	0	n.d.	n.d.	n.d.	PD	3.09	5.59

* In these cases, histological and molecular analyses were performed on primary tumors, in all other cases on recurrent tumors (all pts received temozolomide and RT as first treatment); °: censored; n.d.: not done; SD: stable disease; PD: progressive disease. MMR: mismatch repair; MSI: microsatellite instability; TMB: tumor mutational burden; PFS: progression-free survival; OS: overall survival.

**Table 3:** Pembrolizumab activity.

Complete Response	0/13 (0%)
Partial Response	0/13 (0%)
Stable Disease	4/13 (31%)
Disease Control Rate	4/13 (31%)
Progression Disease	9/13 (69%)
mPFS	2.2 months (95% CI 1.6–2.8)
6 month-PFS	23%
mOS	5.6 months (95% CI 0.1–11.9)
12 month-OS	38%

PFS: progression-free survival; OS: overall survival.

## Supplementary Materials

The following are available online at https://www.mdpi.com/2072-6694/12/8/2283/s1, Figure S1: List of somatic and germline mutations identified in all 12 cases analyzed by next generation sequencing of 409 genes using the TML panel, Table S1: Baseline patient characteristics, Table S2: Patient immune-molecular characteristics, Table S3: Summary of gene alterations identified by sequencing analysis of 12 glioma cases, Table S4: Associations of molecular and immunological variables with disease control rate.
