# Supplementary material for: Pembrolizumab Activity in Recurrent High-Grade Gliomas with Partial or Complete Loss of Mismatch Repair Protein Expression: A Monocentric, Observational and Prospective Pilot Study
Source: Cancers (Basel). 2020 Aug 14;12(8):2283. doi: 10.3390/cancers12082283 (PMC7464918; doi:10.3390/cancers12082283)
Supplement: Supplementary file 1 [file cancers-12-02283-s001.pdf]

Supplement Materials: Pembrolizumab Activity in Recurrent High-Grade Gliomas with Partial or Complete Loss of Mismatch Repair Protein Expression: A Monocentric, Observational and Prospective Pilot Study

Methods S1.

Mutational and Copy Number Variation Status

DNA was obtained from tumor and matched non-neoplastic brain (surrounding the tumor and showing no microscopic neoplastic infiltration) using 10 consecutive formalin-fixed paraffin-embedded (FFPE) 4-μm sections and the QIAamp DNA FFPE Tissue Kit (Qiagen). DNA was qualified as reported elsewhere[1].

Two different targeted next-generation sequencing (NGS) panels were used. The first was the Oncomine Tumor Mutational Load (TML) assay (ThermoFisher), which covers 1.65 Mb of genomic space for the assessment of tumor mutational burden and includes all exons of 409 cancer-related genes for mutational and copy number assessment. This gene panel has been reported to have 0.99 Pearson correlation with whole exome sequencing in the assessment of TMB (Budczies et al. Ann Oncol. 2019; 30(9):1496-1506; PMID: 32119917).The second was a custom panel named ACC GBM panel, which was designed with the contribution of the Italian Alliance Against Cancer (ACC) and explores the mutational asset of 53 glioma-associated genes including: ACVR1, AKT1, ASCL1, ATRX, BRAF, CDK4, CDK6, CDHN2A, CDKN2B, CIC, DAXX, EGFR, EPCAM, FGFR1, FGFR2, FGFR3, FUBP1, H3F3A, IDH1, IDH2, KIAA1549, LTBP4, LZTR1, MDM2, MDM4, MET, MLH1, MLH3, MSH2, MSH4, MSH5, MSH6, MYCN, NDRG1, NF1, NF2, NFKBIA, NOTCH1, PDGFRA, PIKCA, PIKER1, PMS1, PMS2, POLD1, POLE, PTEN, RB1, SMARCA4, SMARCB1, SPRED1, TACC3, TERT, TP53.

Libraries for TML assay were prepared using 20ng tumor DNA and sequenced on the Ion S5XL system (Thermofisher) loaded with Ion 540 chip as described[2]. Reads alignment to the hg19 human reference genome and variant calling, was done using Torrent Suite Software v.5.10 (Termofisher).

Libraries for ACC GBM panel were prepared using 150 ng tumor DNA and the SureSelect XT High Sensitivity protocol (Agilent Technologies, Santa Clara, USA) and sequenced on Illumina MiSeq. Reads alignment to human genome hg19 and variant calling was performed using the OncoKDM algorithm (OncoDNA S.A., Gosselies, Belgium), which allows to identify both single nucleotide substitutions and copy number variations.

Tumor mutational burden and mutational signatures

Tumor mutational burden and mutational spectrum were evaluated using the Oncomine TML 5.10 plugin available on IonReporter software (Thermofisher). Default Modified parameters were used according to manufacturer's protocol in order to exclude false positive results due to sequencing artefacts. A threshold of at least 20 reads and a variant allelic frequency of at least 10% were used to perform mutation calling. Additional technical details have been described elsewhere[2].

Microsatellite instability

Microsatellite instability was assessed using the Titano Kit (DiatechPharmacogenetics), which analyzes 6 poly-A microsatellites (BAT25, BAT26, BAT40, NR21, NR24, TGFβRII) and 4 dinucleotide markers (D2S123, D17S250, D5S346, D18S58).

MGMT methylation status

MGMT promoter methylation status was analyzed by pyrosequencing using a commercial kit (MGMT plus, Diatech Pharmacogenetics) on a PyroMark Q96 ID system equipped with PyroMark CpG (Qiagen) software.

PD-L1 and MHC-1 immunohistochemistry

Immunohistochemistry with anti-PD-L1 (clone 22C3; Dako) and anti-MHC class I (clone ES05; Dako) primary antibodies was performed using the BOND-MAX system (Leica Biosystems). Immunostainings were jointly assessed by two pathologists.

Macrophage and CD8+ cell density analyses

A multispectral imaging analysis was performed on 4μm FFPE tissue slides, using three markers: CD8 (clone C8/144B, Termofisher Scientific), which recognizes cytotoxic T lymphocytes, CD68 for the total macrophage fraction (clone PG-M1, Dako Agilent), and glial fibrillary acidic protein GFAP (clone EP672Y, AbCam) as a tissue architecture marker. The DAPI (Spectral Dapi, Akoya Biosciences) nuclear counterstain was optimized in order to gain a multicomponent view of the cellular infiltrate. The stained tissue slide was accompanied by an unstained control slide to subtract the background tissue autofluorescence signal. Images were acquired with the Mantra multispectral imaging platform (Akoya Biosciences) at 20x magnification. Analysis was performed using InForm 2.4.1 software (Akoya Biosciences). Cell density/mm^2 was chosen as the election parameter to quantitively characterize the immune cell infiltrate.

References

1. Simbolo, M.; Gottardi, M.; Corbo, V.; Fassan, M.; Mafficini, A.; Malpeli, G.; Lawlor, R.T.; Scarpa, A. DNA qualification workflow for next generation sequencing of histopathological samples. *PloS One* **2013**, *8*, e62692. doi:10.1371/journal.pone.0062692
2. Barresi V, Simbolo M, Mafficini A, et al. Ultra-Mutation in IDH Wild-Type Glioblastomas of Patients Younger than 55 Years is Associated with Defective Mismatch Repair, Microsatellite Instability, and Giant Cell Enrichment. *Cancers* **2019**, *11*, doi:10.3390/cancers11091279

Table S1. Baseline patient characteristics.

Characteristics	N (%)
Patients	13
Median Age	43(range 21-65)
Gender	
Male	7 (54)
Female	6 (46)
ECOG PS	
0	3 (23)
1	9 (69)
2	1 (8)
Histology	
Anaplastic Astrocytoma	4 (31)
Anaplastic ODG	1 (8)
Glioblastoma	8 (61)
Surgery at recurrence	9 (69)
Prior RT	13 (100)
Median previous CT lines	2 (range 1-5)
Temozolomide	13 (100)
Steroids	
Yes	5 (38)
No	8 (62)
MGMT status	
Methylated	9 (69)
Unmethylated	4 (31)

IDH status	
Wild-type	9 (69)
Mutated	4 (31)

ECOG PS: Eastern Coopertaive Oncology Group Performance Status; ODG: oligodendroglioma; RT: radiation therapy; CT: chemotherapy.

Table S2. Patient immune-molecular characteristics.

Characteristics	N (%)
Status of MMR protein expression	
Complete loss of MSH6+MSH2	1 (8)
Complete loss of MSH6	1 (8)
Partial loss of MSH6+MSH2	6 (46)
Partial loss of MLH1+PMS2	2 (15)
Partial loss of MSH6	1 (8)
Partial loss of MSH2	2 15)
Tumoral MMR gene mutations	
MSH6	1 (8)
Microsatellite Status	
Stable (MSS)	13 (100)
Median PD-L1 expression	0% (range 0-5)
Median TMB*	10.02 mutations/megabase (range 6.8-26.89)
Median MHC-1 expression*	60% (range 0-95)
Median macrophage density*	423.85/mm^2 (range 108.9-971.2)
Median CD8$^+$ density*	25.9/mm^2 (range 0-90.7)

MMR: mismatch repair; MSS: microsatellite stable: TMB: tumor mutational burden; * analyzed in 12 pts

Table S3. Summary of gene alterations identified by sequencing analysis of 12 glioma cases.

Gene	*n*	%	M	N	F	S	A	HD
AKT2	1	8,3			1			
ATRX	1	8,3			1			
CDK4	1	8,3					1	
CDKN2A/B	6	50,0						6
EGFR	2	16,7					2	
FGFR2	1	8,3	1					
IDH1	4	33,3	4					
KDR	1	8,3					1	
KIT	1	8,3					1	
MDM4	1	8,3					1	
MSH6	1	8,3			1			
NF1	2	16,7	1			1		
PDGFRA	1	8,3					1	
PIK3C2B	1	8,3					1	
PIK3R1	1	8,3		1				
PIK3R2	1	8,3	1					
PMS1	1	8,3	1					
PTEN	1	8,3	1					
PTPN11	2	16,7	2					
RB1	1	8,3		1				
RET	2	16,7	2					
TP53^	8	66,7	8		1			

Note: n: number cases affected; %: mutation rate estimated on 12 cases; M: missense mutation; N: nonsense mutation; F: frameshift; S: splice site alteration; A: amplification (>4 gene copies); HD: homozygous deletion. ^ One cases showed biallelic missense mutation.

Table S4. Associations of molecular and immunological variables with disease control rate.

**Variable**	***p* Value**
Loss of (yes vs no)	
MSH2	0.7
MSH6	0.4
PMS2	0.5
MLH1	0.5
MMR protein loss (complete vs partial)	0.9
MGMT status (met vs unmet)	0.2
IDH status (mut vs wt)	0.9
TP53*	0.6
CDKN2A/B*	0.7
NF1*	0.2
TMB*°	0.5
MHC-1 expression*°	0.9
Macrophage density*°	0.9
$CD8^+$ density*°	0.9

*analyzed in 12 patients; comparison of median values between group of patients with stable disease and progressive disease (Mann-Whitney tests).

ID	GENE	CHROM	POS	REF	ALT	var_freq	Status	Existing_variation	Feature	Consequence	HGVSc	HGVSp	EXON	INTRON	CLIN_SIG
1PD	*TP53*	chr17	7577121	G	A	63	somatic	rs121913343	NM_000546.5	missense_variant	NM_000546.5:c.817C>T	NP_000537.3:p.Arg273Cys	8/11		pathogenic
2PD	*TP53*	chr17	7577536	T	C	19	somatic	rs587782082	NM_000546.5	missense_variant	NM_000546.5:c.745A>G	NP_000537.3:p.Arg249Gly	7/11		likely_pathogenic
2PD	*NF1*	chr17	29509525	G	T	12	somatic	COSM6910666	XM_005257983.1	splice_acceptor_variant	XM_005257983.1:c.731-1G>T			7/58	
3PD	*TP53*	chr17	7576896	TG	T	87	somatic	COSM10786	NM_000546.5	frameshift_variant	NM_000546.5:c.949del	NP_000537.3:p.Gln317SerfsTer28	9/11		
3PD	*NF1*	chr17	29653118	CTTGT	TTTGT	86	germline	rs769087878	XM_005257983.1	missense_variant	XM_005257983.1:c.5116delinsT	XP_005258040.1:p.Leu1706Phe	37/59		
3PD	*RB1*	chr13	48955394	C	T	64	somatic	rs886043247	NM_000321.2	stop_gained	NM_000321.2:c.1510C>T	NP_000312.2:p.Gln504Ter			pathogenic
4PD	*TP53*	chr17	7577094	G	A	21	somatic	rs28934574	NM_000546.5	missense_variant	NM_000546.5:c.844C>T	NP_000537.3:p.Arg282Trp	8/11		pathogenic
5PD	*IDH1*	chr2	209113113	G	T	27	somatic	rs121913499	NM_001282387.1	missense_variant	NM_001282387.1:c.394C>A	NP_001269316.1:p.Arg132Ser	4/10		pathogenic
5PD	*TP53*	chr17	7577091	G	A	51	somatic	rs149633775	NM_000546.5	missense_variant	NM_000546.5:c.847C>T	NP_000537.3:p.Arg283Cys	8/11		likely_pathogenic
5PD	*TP53*	chr17	7578266	T	A	25	somatic	rs942158624	NM_000546.5	missense_variant	NM_000546.5:c.583A>T	NP_000537.3:p.Ile195Phe	6/11		likely_pathogenic
5PD	*ATRX*	chrX	76939464	ATTTTTC	ATTTT	31	somatic		NM_000489.3	frameshift_variant	NM_000489.3:c.1278_1279del	NP_000480.2:p.Asn428TyrfsTer5	9/35		
9PD	*IDH1*	chr2	209113112	C	T	31	somatic	rs121913500	NM_001282387.1	missense_variant	NM_001282387.1:c.395G>A	NP_001269316.1:p.Arg132His	4/10		pathogenic
9PD	*TP53*	chr17	7578271	T	C	77	somatic	rs786201838	NM_000546.5	missense_variant	NM_000546.5:c.578A>G	NP_000537.3:p.His193Arg	6/11		likely_pathogenic
9PD	*ATRX*	chrX	76888721	ATTTC	A	32	somatic	COSM1716708	NM_000489.3	frameshift_variant	NM_000489.3:c.5104_5107del	NP_000480.2:p.Glu1702TyrfsTer22	19/35		
9PD	*PMS1*	chr2	190708712	G	A	41	germline	rs2066459	XM_005246648.1	missense_variant	XM_005246648.1:c.605G>A	XP_005246705.1:p.Arg202Lys	6/13		uncertain_significance
10PD	*PTEN*	chr10	89692917	T	C	50	somatic	rs1085308046	NM_000314.4	missense_variant	NM_000314.4:c.401T>C	NP_000305.3:p.Met134Thr	5/9		pathogenic
12PD	*PTPN11*	chr12	112888165	G	T	45	germline	rs397507510	NM_001330437.1	missense_variant	NM_001330437.1:c.181G>T	NP_001317366.1:p.Asp61Tyr	3/16		pathogenic
13_1PD	*RET*	chr10	43607561	GC	GG	50	germline	rs149238501	NM_020975.4	missense_variant	NM_020975.4:c.1538delinsG	NP_066124.1:p.Ala513Gly	8/20		uncertain_significance
16PD	*IDH1*	chr2	209113112	C	T	60	somatic	rs121913500	NM_001282387.1	missense_variant	NM_001282387.1:c.395G>A	NP_001269316.1:p.Arg132His	4/10		pathogenic
16PD	*TP53*	chr17	7577538	C	T	91	somatic	rs11540652	NM_000546.5	missense_variant	NM_000546.5:c.743G>A	NP_000537.3:p.Arg248Gln	7/11		pathogenic
17PD	*AKT2*	chr19	40742182	CG	T	31	somatic		NM_001626.4	frameshift_variant	NM_001626.4:c.941_942delinsA	NP_001617.1:p.Pro314GlnfsTer30	10/14		
17PD	*FGFR2*	chr10	123246934	C	T	67	somatic		NM_022970.3	missense_variant	NM_022970.3:c.1994G>A	NP_075259.4:p.Arg665Gln	15/18		
17PD	*MSH6*	chr2	48027965	AAC	A	60	somatic		NM_000179.2	frameshift_variant	NM_000179.2:c.2845_2846del	NP_000170.1:p.Gln949GlufsTer16	4/10		
17PD	*PIK3R2*	chr19	18273784	G	A	18	somatic	rs587776934	NM_005027.3	missense_variant	NM_005027.3:c.1117G>A	NP_005018.1:p.Gly373Arg	10/16		pathogenic
17PD	*PTPN11*	chr12	112884103	G	A	22	somatic	COSM430369	NM_002834.3	missense_variant	NM_002834.3:c.38G>A	NP_002825.3:p.Gly13Asp	2/16		
17PD	*TP53*	chr17	7577538	C	T	31	somatic	rs11540652	NM_000546.5	missense_variant	NM_000546.5:c.743G>A	NP_000537.3:p.Arg248Gln	7/11		pathogenic
17PD	*PIK3R1*	chr5	67522831	C	T	29	somatic		NM_181523.2	stop_gained	NM_181523.2:c.328C>T	NP_852664.1:p.Gln110Ter	2/16		
18PD	*IDH1*	chr2	209113112	C	T	12	somatic	rs121913500	NM_001282387.1	missense_variant	NM_001282387.1:c.395G>A	NP_001269316.1:p.Arg132His	4/10		pathogenic
18PD	*RET*	chr10	43620335	C	T	49	germline	rs17158558	NM_020975.4	missense_variant	NM_020975.4:c.2944C>T	NP_066124.1:p.Arg982Cys	18/20		pathogenic

Figure S1. List of somatic and germline mutations identified in all 12 cases analyzed by next generation sequencing of 409 genes using the TML panel.
